# The continued retreat of non-profit housing providers in the Netherlands

**DOI:** 10.1007/s10901-015-9458-1

**Published:** 2015-07-22

**Authors:** Nico Nieboer, Vincent Gruis

**Affiliations:** Faculty of Architecture and the Built Environment, Delft University of Technology, P.O. Box 5043, 2600 GA Delft, The Netherlands

**Keywords:** Organisational strategy, Non-profit, Social housing, The Netherlands

## Abstract

After the abolishment of object subsidies for housing construction and renovation in the mid 1990s, Dutch housing associations, the main non-profit housing providers in the country, heavily relied on market activities, such as selling homes to owner occupiers, to generate income for their social activities and to contribute to urban development policies. This worked well, which was one of the main reasons that these housing providers could adopt a wide field of operations, including not only the management and development of affordable housing for low-income groups, but also housing in other market segments, plus activities regarding care, welfare, local economy, employment and education. Recent economic and political developments, however, have caused housing associations to return on this path. Central in this paper is a research among Dutch housing associations about their values, strategic positioning and strategies. The research was executed in two waves (conducted in 2010/2011 and in 2013/2014, respectively), each consisting of a panel survey and interviews with selected panellists. This paper presents the results of the second wave. It is expected that after the first wave of the research, new regulations, such as the national implementation of European rules on state support and the introduction of a new property tax, have resulted in a further retreat from non-social housing activities. The analysis shows that this is indeed the case, but that the main shifts in priorities have not taken place directly after the credit crunch, but in later years.

## Introduction

Since the late 1980s, the non-profit housing sector in the Netherlands shifted from being driven by government regulation and public financing to a sector that had to stand on its own two feet. Capital market loans were introduced into the sector, supported by a joint government and sector-backed loan guarantees, and direct subsidies for new housing supply were largely abolished. Retained regulation took the form of supervision of the operation of housing associations on the basis of general “fields of performance”, regulation of rent increases and limited reporting to supervising bodies. Within this framework, housing associations were free to sell, invest and decide how to allocate their resources to their social task.

The financial liberalisation of the Dutch social housing sector was accompanied by an involvement in both new housing activities and other products and services. In the housing domain, not only did housing development and neighbourhood renewal have more prominent roles, but the sale of both new and existing homes became more important as a means of financing housing development and as a vehicle for cross-subsidising social activities. Organisations also embarked on activities in the field of welfare, care, local economy and education, albeit usually via a restricted role as social real estate developers and facilitators rather than direct providers of broader services (Brandsen et al. 2006; Gruis 2008; Van Overmeeren and Gruis 2011). Not only did the housing associations choose to adopt a wide range of activities, they were also encouraged to do so by the political environment, which demanded more actions in the social sphere.

In the last few years, five developments brought about new dynamics in the Dutch housing sector. First, the credit crunch that followed the global financial crisis (GFC) in 2008 severely affected the housing market and led to restrictions in housing construction initiatives by housing associations as well as sharp falls in the asset values of their property and land portfolios. It also triggered more cautious bank lending policies, which reduced the capacity of housing associations to invest, thus also contributing to a sharp decline in their housing turnover (Priemus 2010). Second, the national government abolished the exemption from corporate tax for non-profit activities, which also reduced their investment opportunities. Third, the market privileged position of housing associations became a bone of contention with the private sector, leading to interventions under a European competition directive. The Dutch Government’s implementation of EU regulations on state aid entailed a considerable change in the allocation of homes, with the result that housing associations now have to focus more on assisting low-income households than they used to do in the past, in order to remain eligible for state support (Priemus and Gruis 2011). Fourth, a new property tax was introduced in 2014 for homes in the regulated rent segment. The revenue yielded by this tax was estimated to increase from €1.1bn (€541 per home on average) in that year to €1.7bn (€775 per home on average) in 2017 (www.aedes.nl). Finally, during the second wave of the research, intensive debates were held about a reform of the Housing Act, including a considerable restriction of the housing associations’ activities, especially those outside the traditional area of lower-income housing. Although the new Housing Act has only been in force since 2015, it can be expected that these debates have already had an effect on the housing associations, as these organisations anticipated the new law.

It is plausible that developments like these have had a profound effect on the investment behaviour and related strategies of individual organisations in the social housing sector. We carried out a survey in 2010, followed by an interview round in 2011, to establish to what extent this expectation was true (reports on this in Nieboer and Gruis 2011, 2012, 2014). The results of that research indicated a strong shift in behaviour in the years before, which can be summarised as a shift from a wide field of activities in both the housing and the non-housing sphere towards a focus on core business activities, namely providing affordable housing for low-income households. The research also indicated that this shift would continue in later years. In 2013/2014, we repeated the study to test the validity of this expectation and, more in general, to identify some trends that emerged since the last data collection. This paper primarily presents the results of this second wave. As in the first wave, we used a classification of organisational strategies developed by Gruis (more details about this model are given in Sect. 2).

The research questions addressed in this paper are:

- What are the current values and strategic priorities of Dutch housing associations?
- To what extent do these values and priorities differ from those of the recent past?
- What strategies are Dutch housing associations expected to pursue in the near future?
- In which sense and to what extent do these strategies and activities differ from those of the recent past?
- To what extent can developments in organisational strategies be classified using Gruis’ model?

The paper is structured as follows. In Sect. 2 the context and the theoretical framework, including Gruis’ model, are addressed, while the research approach and the research methods are described in Sect. 3. Section 4 presents the results of the second wave of the research, which are compared with those from the first wave in Sect. 5. Finally, conclusions are drawn in Sect. 6, where we also provide some ideas for further research in this field.

## Context and theoretical framework

Like many other non-profit organisations, housing associations can be regarded as “hybrid” entities. In this respect, the hybridity refers to the fact that these organisations have to act in a “tension field” (Mullins et al. 2014) between government, market and community, and have to cope with the different and often competing interests and drivers from each of these three entities (Brandsen et al. 2005; Mullins et al. 2012; Blessing 2012, 2014). The position of housing associations in between these entities is not constant, but rather varies with time. In the last 25 years, we witnessed a massive privatisation of the non-profit housing sector in Eastern Europe and, to a smaller extent, in the UK. In the same period, non-profit housing providers in several Western European countries (e.g. the Netherlands, Austria and again the UK) and Australia moved away from government control and support and were driven towards increased market orientation. Owing to this development, non-profit housing providers became conscious of their autonomy and positioned themselves at even further distance from government by, for example, engaging in project development activities in commercial housing (for rent and for sale) and by diversification into non-housing activities (Table 1). As has been stated in the introduction, political developments and unfavourable market circumstances in the last years caused housing associations to once again find a position between government, market and community. In terms of housing investments and disinvestments, the most visible decline can be observed in production for sale. In addition, a reduction in project development activities resulted in a lower number of demolitions.

**Table 1:** Changes in the number of housing units in the Dutch social housing sector

Years	2007	2008	2009	2010	2011
New build for rent	25,200	28,000	30,400	28,600	28,600
New build for sale	8500	8500	10,000	7900	6700
Purchase from outside the sector	2900	4800	4400	2200	2800
Sale to households	14,300	13,400	13,100	15,100	14,300
Sale to entities other than households outside the sector	700	800	300	400	3800
Demolition	15,900	16,000	15,800	13,100	11,900

*Source*: BZK (2013, p. 165). For comparison, the total number of housing units in the sector in this period was around 2.4 million

The continuing shift in changing priorities is also reflected in the investment prognoses of housing associations, as can be seen in Table 2.

**Table 2:** Investment prognoses of all housing associations (in number of housing units) for three periods

Years	2008–2012	2010–2014	2012–2016
New build for rent	230,000	175,100	148,700
New build for sale	110,000	74,600	44,000
Sale to households	86,200	91,500	88,200
Demolition	111,700	85,000	61,500
Refurbishment^a^	92,500	105,700	113,400
Investment in non-residential real estate with social purpose^b^	n.a.	€2.9bn	€1.8bn

*Source*: Central Housing Fund

^a^Investment above €20,000 per dwelling

^b^e.g. schools, community centres

The tabulated figures show a clear shift from new housing development and demolition to refurbishment. Furthermore, the level of investment in non-residential real estate for social purposes has been expected to decline significantly.

**Fig. 1:**
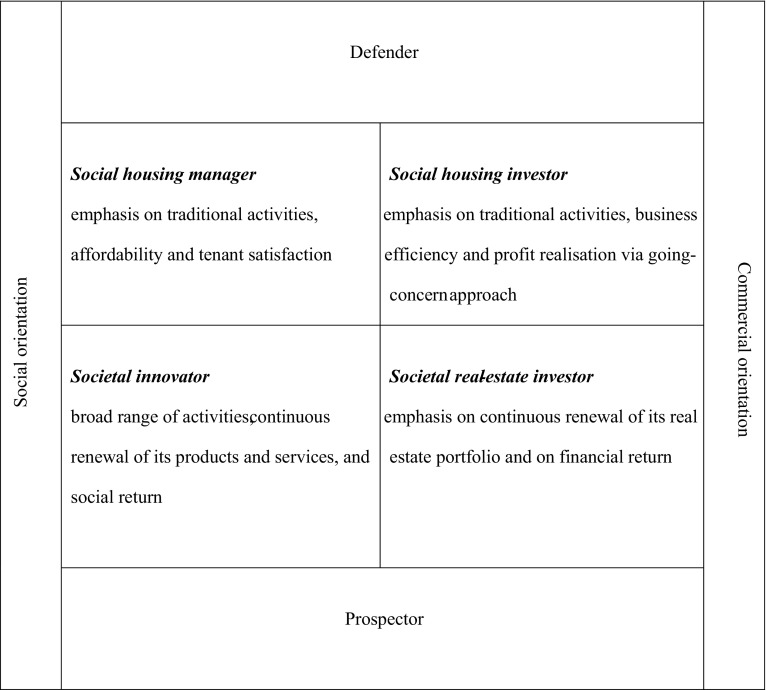
Conceptual organisational archetypes of housing associations. *Source*: Gruis 2008 (adapted by the authors)

To analyse the shift that has occurred in the priorities and activities of Dutch housing associations, we have used Gruis’ description of organisational archetypes, which are extensively addressed by Gruis (2008) and are also explained in a publication pertaining to the first wave of this research (Nieboer and Gruis 2014). In this typology, the prospector–defender dimension is combined with the social–commercial dimension to define four organisational archetypes (see Fig. 1):

- *Social Housing Manager*: Emphasises traditional social housing tasks, strives to deliver services to clients efficiently and primarily focuses on achieving social returns and actively uses its financial surpluses in the interest of housing;
- *Social Housing Investor*: Emphasises traditional activities, strives for business efficiency and generates financial returns by adopting a ‘going-concern’ approach to managing the housing stock;
- *Societal Innovator*: Undertakes a broad range of work in terms of both market segments and public sectors, strives to continuously develop this field of work and the products and services provided, focuses primarily on social returns, and actively uses its financial surpluses in the interest of society;
- *Societal Real Estate Investor*: Undertakes mainly activities on the real estate market, strives to continuously (re)develop its real estate portfolio and predominantly focuses on achieving a real estate portfolio that can provide good financial returns, while taking into account its social (housing) objectives.

The typology is based partly on Miles and Snow’s (1978) distinction between prospectors and defenders. Prospectors are organisations that are trying to secure their continuity through continuous market development and expansion; defenders are organisations that emphasise efficiency and a competing price–quality range in a limited, fixed market domain. Additionally, Gruis’ typology is inspired by Walker (2000), who posits that tensions between property and welfare approaches in social housing provision are likely to lead to the domination of a property-based approach because of on-going managerial and external pressures on social housing organisations. Nevertheless, Walker also suggests another scenario, whereby the development and enhancement of a “housing plus agenda” would turn associations into organisations where property management is only one aspect of their work. Support for both scenarios in the Dutch as well as UK context can be found in several studies, including Clapham and Satsangi (1992), Kemp (1995), Symon and Walker (1995), Walker (1994, 2000), Nieboer and Gruis (2014) and Brandsen et al. (2006).

Gruis’ typology has been applied in several studies in the Netherlands, the UK and Australia (e.g. Gruis 2008; Milligan et al. 2012; Morrison 2013; Nieboer and Gruis 2014; Czischke 2014) as a framework for analysing strategic priorities of not-for-profit housing providers. In this article, we employ it as a framework to analyse recent developments in the Dutch housing association sector. Our assumption is that, while housing associations’ priorities respond to general contextual developments in policy and economy, these responses are also “filtered” by values of housing associations’ management, local market and policies, available resources and path dependency (e.g. Walker 2000; Sacranie 2012; Czischke 2014). Given the contextual developments described in the introduction of the article, we expect that the increasingly shrinking budgets of Dutch housing associations have led to a further retreat to core business. This shift will particularly be evident as a result of the substantial levy laid upon housing associations by the current government (which is much higher that the levy announced by the previous government), combined with the continued difficulties in the housing market, which limit the potential for generating additional revenue through the sale of homes. Additionally, the current Housing Minister’s proposed amendment of the Housing Act to place substantial restrictions on financing developments of more expensive rental dwellings and owner-occupied housing will lead housing associations to become even more restrictive in carrying out these activities.

However, we also expect this retreat to core business to be slower in the last 3 years (between 2010 and 2013) than in the years immediately following the GFC, assuming that the main reduction in the scope of priorities has already taken place and that housing associations have already balanced their priorities in favour of their core businesses.

## Research approach

In this research, we employed a modified Delphi technique, originally applied by Mullins (2006) for a study in England guided by largely similar research questions. In his study, it was decided not to distribute a questionnaire among a statistically representative group of housing associations, but to select a smaller group of organisations for a more in-depth investigation which consisted of a survey plus subsequent interviews with some of the survey respondents. As in the English study, a survey was drafted for the Dutch research on the basis of propositions and questions that were derived from the analytical framework, as described in the previous section. The goal was to identify changes that have taken place in housing associations’ operations in the Netherlands. Most answers could be given on a seven-point scale. Respondents were sometimes asked to indicate their perceptions of the situation 3 years previously (i.e. in 2010), even though some authors (e.g. Golden 1992) argue that such a retrospective approach may not produce reliable outcomes because answers concerning priorities in the past can be subject to biases as a result of current priorities. Our method, however, enabled us to obtain comparable data concerning both past and current priorities. Moreover, we deemed some level of bias tolerable, since our main objective was to shed light on shifts in priorities.

The housing associations were asked to have the questionnaire completed by someone with a good overview of the organisational policies, the motives behind these policies, and who would be able to provide insight into the expected strategic direction of the organisation in the near future. This person may be a director, a member of the management team or a senior policy officer. Because the respondents were also asked to give their job title, we were able to verify whether the housing associations had complied with this request. In general, this proved to be the case, with the possible exception of a few cases in which the wording used left some doubt.

When selecting organisations for inclusion in the study, we opted for an intensive telephonic preparation among a group that had a relatively limited size, but was sufficiently large to allow us to make indicative statements about the sector as a whole. This telephonic preparation enabled us to identify the right staff member to complete the questionnaire and gave us the opportunity to motivate him/her to participate. Housing associations were selected from the professional network of the researchers and their colleagues. We decided to limit our sample to housing associations with their own policy staff in order to reduce the risk of the strategic priorities of the organisation not being documented or otherwise articulated. Housing associations that had merged during the 2 years prior to the survey were excluded, because it was expected that the policy of such new organisations would still be in flux to some extent.

For the first wave in 2010, 41 housing associations were approached to participate in the survey; 31 returned the completed questionnaire. The response then contained sufficient variation in terms of regional origin, financial situation and investment obligations (notably restructuring activities). However, partly due to this selection method, large- and medium-sized housing associations (defined here as all organisations that own more than 4000 homes) were overrepresented in the responses. In the first wave of the research, a relatively high number of the organisations were active in the Randstad (13 out of 31), an urban area in the west of the Netherlands that includes the four largest cities in the country.

For the second wave, the 31 respondents that took part in the first wave were approached again. The questionnaire utilised in this wave was distributed in November 2013 and was completed by 23 housing associations. While the overrepresentation of the large and medium-sized housing associations persisted (as expected, due the initial sample structure), the institutions were otherwise evenly distributed in terms of region, financial situation and investment obligations.

In the first wave of the research, after the survey (in November and December 2011), interviews were held with ten housing associations in order to explore the motivation for their answers in the questionnaire. The selection was made by choosing every third organisation from an alphabetical list of the 31 responding housing associations. The interviews were held with the same individuals who had filled in the questionnaire. In the second wave, 12 such interviews were held, in February and March 2014. The selection was made by choosing every second organisation from an alphabetical list of the 23 responding housing associations. Alignment with similar research in some other countries was the main reason for having slightly more interviews in the second wave than in the first one.

## Findings: results of second wave

In the questionnaire the following topics were addressed:

- the values of the organisation;
- its strategic priorities;
- the perceived changes and their influence on the operational activities of the organisation;
- the strategies (in terms of actions and activities) of the organisation.

The results pertaining to each of these topics will be presented in the subsequent sections. Although the respondents were also asked to indicate their organisation’s main strategic decisions for the next few years, this subject is discussed in Sect. 5, together with the results of the previous wave.

### Balance of values

**Fig. 2:**
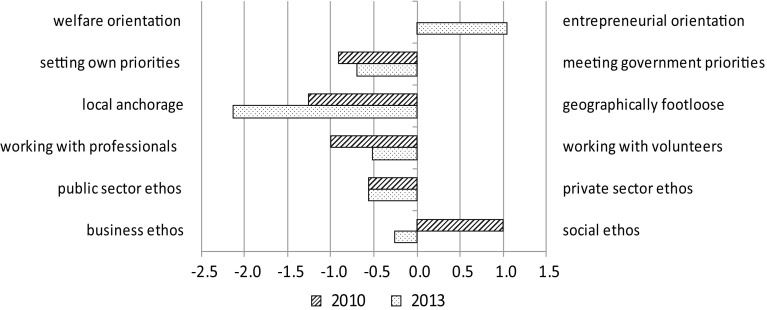
Average balances of pairs of values, in 2010 and in 2013. In 2010 the average “weight” for the welfare orientation and the entrepreneurial orientation was exactly the same. For this reason, no *bar* is depicted for this pair of values

In the questionnaire, respondents were presented with pairs of possible values and asked to indicate to what extent one priority took precedence over the other for their particular organisation, as well as what the situation had been 3 years earlier. Answers were given on a seven-point scale, which were converted into corresponding numbers ranging from −3 to +3. The lower the number, the more the emphasis on the priority on the left-hand side; the higher the number, the more the emphasis on the priority on the right-hand side, with 0 indicating that the two priorities had equal emphasis. Figure 2 gives the average score for each pair of values, showing the extent to which one priority predominated over the other in the group of associations as a whole.

For 2010, it can be observed that local anchorage was perceived as more important than being geographically footloose, working with professionals took precedence over working with volunteers, and the social ethos dominated the business ethos. In 2013, with the exception of local anchorage, these preferences are less clear. The interviews held after the survey show that the declining popularity of geographical expansion is the main reason for this dominance. This, in turn, is likely due to shrinking budgets and, related to that, decreased investment capacities.

In general, a more businesslike attitude can be observed in the sector: the relative importance of the social ethos has decreased for the “benefit” of the business ethos, and the entrepreneurial orientation has gained importance compared with the welfare orientation.

### Balance of strategic priorities

**Fig. 3:**
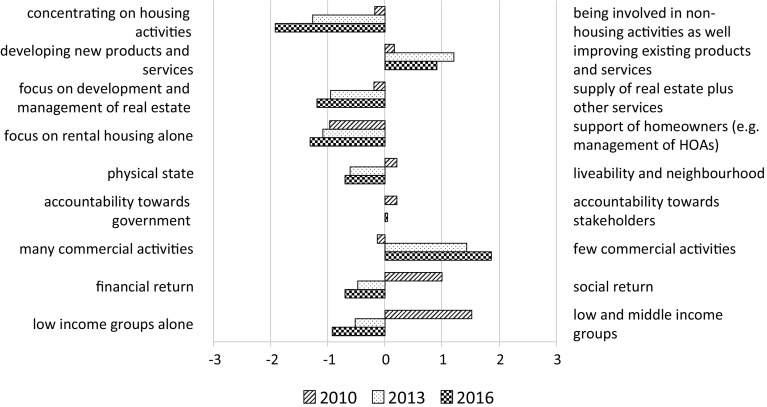
Average balances of pairs of possible priorities, in 2010, 2013 and 2016

Respondents were again presented with pairs, now of possible strategic priorities, and were asked to compare the situation at the time of the survey (2013) with that of 3 years earlier (2010). They were also asked to give an expectation for 2016. Answers were again given on a seven-point scale, which was converted into a number ranging from −3 to +3. Figure 3 gives the average score for each pair of strategic priorities, thus showing the extent to which one priority predominated over the other in the group of associations as a whole.

The developments in the years 2010–2013 clearly indicate a movement in the sector towards the traditional role of housing manager: less involvement in non-housing activities, less emphasis on the development of new products, more focus on the development and management of real estate, more focus on the physical state of the housing stock, fewer commercial activities and more emphasis on low-income groups alone. As one of the interviewees stated:

Until only a few years ago we invested a lot in activities other than social housing alone. (…) We developed a multitude of plans, even for the acquisition of [commercially operated] parking facilities. This has all been abandoned. Now, we intend to dispose of all our commercial, non-housing real estate, unless it has a function for the neighbourhood or generates a sound return.

The interviews show what housing associations see as the main causes, namely the current economic crisis, a larger tax burden and stricter allocation rules, the latter being a consequence of the Dutch implementation of European regulations regarding state support. The expectations for 2016 point to a further development in these directions, although changes relative to 2013 are rather small. Apparently the housing associations expect that the greatest part of the residualisation of the sector has already taken place.

The data presented here prompt the question of how a return to the traditional, non-commercial tasks can be combined with an increase in a more businesslike ethos in the sector, as indicated in Fig. 2. The interviews indicate that such an ethos must be understood here as an increased attention to efficiency and expenditure savings, which is of great importance in times of declining investment budgets.

### Influence of external changes

**Fig. 4:**
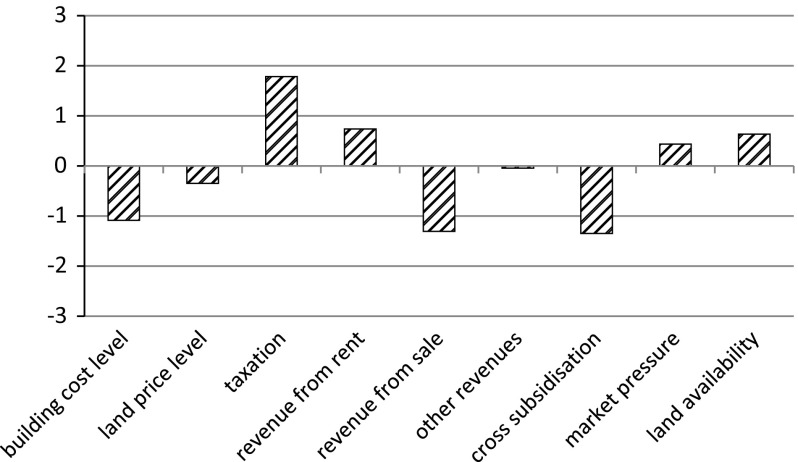
Extent and directions of external changes

The questionnaire also inquired into of external changes, notably the size of these changes and their influence on how the organisation has to fulfil its tasks. The aim of these questions was to identify the changes that the housing associations perceive as the main ones. The respondents were also asked to indicate the direction of some of these changes (increase/decrease). In these cases the responses were also given on a seven-point scale. However, as it was used in a different way, we deal with them separately from the other cases. Figure 4 shows the changes and their respective directions.

The rise in taxation clearly stands out as the most prominent change. Relatively big decreases are also perceived for the building cost level, the revenue from the sale of homes and cross-subsidisation. On average, the housing associations that took part in the research hardly perceive any change in revenues other than those yielded by rent and home sales. For this reason, no bar is depicted at this point.

**Fig. 5:**
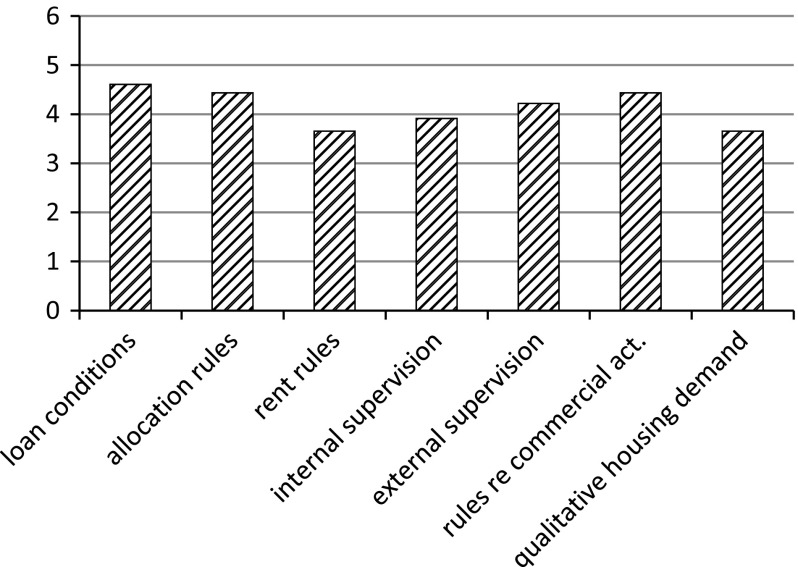
Extent of external changes

Figure 5 shows the size of the changes pertaining to other subjects addressed in the questionnaire.

Among the subjects presented in the figure, the terms and conditions for obtaining a loan, rules for the allocation of homes and rules regarding commercial activities were the areas where the surveyed housing associations perceived the greatest changes. The terms and conditions for obtaining a loan, rules for the allocation of homes and rules regarding external supervision were seen as the most influential changes for the organisation (not depicted). The interviews revealed that many respondents interpreted the change in regulations regarding supervision not primarily as a change in the rules as such, but rather as a shift in the administrative burden imposed on them by regulatory bodies (e.g. extra financial reporting). As one director put it:

There are several new tests and forms. All this financial reporting consumes much more time than in the past. I can almost allocate an extra full-time employee to this work.

### Importance of strategies

**Fig. 6:**
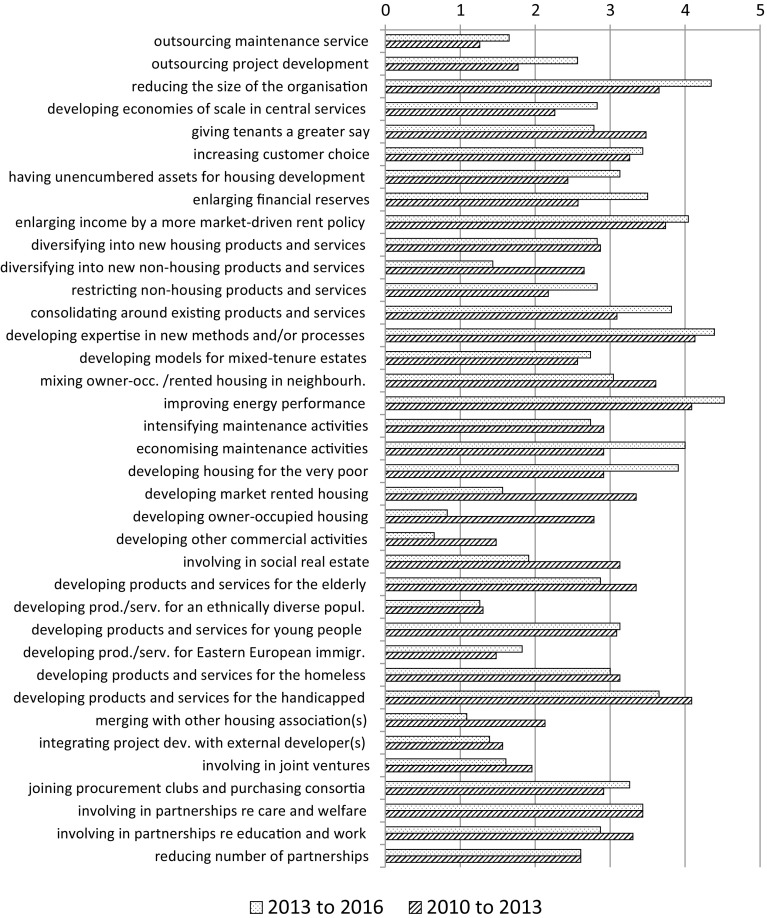
Importance of strategies over the periods 2010–2013 and 2013–2016

The respondents were presented with a number of strategies and were asked to indicate their importance to their association over the last 3 years, as well as their expected importance in the next 3 years. They were instructed to indicate strategies that are/will be important to the association (the actual situation), rather than state their view of which strategies ought to be important (the desired situation). Again, the respondents gave their answers on a seven-point scale, converted to a range from 0 = “very unimportant” to 6 = “very important”. In other words, the higher the number the more important the strategy is/will be to the association(s) in question. If a particular strategy had not been adopted at all, respondents were asked to indicate that it was “very unimportant” to their association. Figure 6 presents the results for the 3 years preceding the survey (2010–2013) and the subsequent three-year period (2013–2016).

The five strategies that the housing associations that took part in the research regarded as the most important ones in the period 2010–2013 were:

- reducing the size of the organisation;
- enlarging income by a more market-driven rent policy;
- improving the energy performance of the housing stock;
- developing expertise in new construction methods and/or processes;
- developing products and services for the physically and/or mentally handicapped.

These strategies are almost the same as the five most important strategies mentioned for the period 2013–2016. The only exception is the strategy regarding the handicapped, which was replaced by economising maintenance activities.

These five most important strategies show a mixed picture. While they indicate the emphasis on reinforcing the financial situation (reducing the size of the organisation, enlarging income by a more market-driven rent policy), they also entail higher investments (improving the energy performance, developing expertise). Nevertheless, strategies pointing to a restriction to fewer types of activities are generally more dominant in the coming 3 years than they were in the preceding 3-year period. Developing housing outside the traditional market for housing associations, namely that for low-income households, has clearly lost importance, while changing the size and the structure of the organisation has gained importance.

## Findings: comparison with the first wave

In this section, some results from the second wave of the research are compared with those from the first wave, with the emphasis on the speed of the changes in each of these periods. In other words, the aim is to establish whether the shifts between 2010 and 2013 are larger or smaller than those between 2007 and 2010.

Further, we reveal what housing associations perceive as their most important strategic decisions for the upcoming period. As has been stated in the introduction, all 31 housing associations that took part in the first wave were approached for the second wave, in which 23 took part. In this paper, we compare the results from the 23 respondents in the second wave both with the same 23 respondents in the first wave and with the entire group of 31 respondents in that wave.

### Shifts in strategic priorities

**Fig. 7:**
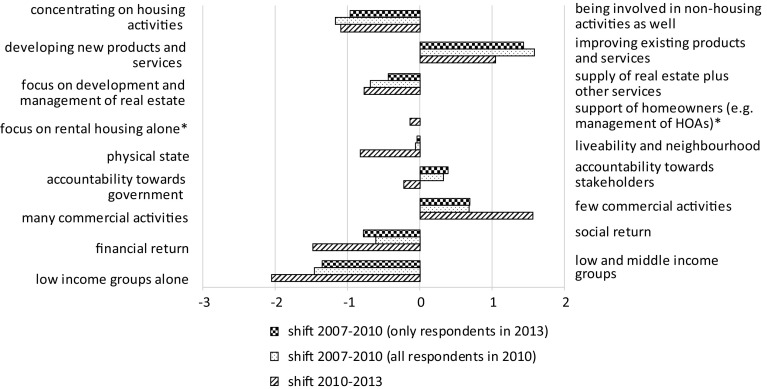
Shifts in balance of pairs of strategic priorities between 2007 and 2010 and between 2010 and 2013. *This item was not included in the survey held in 2010. For this reason, only a *bar* for the shift between 2010 and 2013 is depicted

First we compare the shifts in balance of pairs of strategic priorities between 2007 and 2010 and between 2010 and 2013. These shifts are depicted in Fig. 7.

Most shifts in 2010–2013 are generally perceived as bigger than in 2007–2010, irrespective of whether all respondents to the 2010 survey or only the respondents among them that also participated in 2013 are taken into account. Regarding the focus on low-income groups alone the increased shift is plausible, because the new allocation rules became effective in 2011. For the other items, however, the increased shifts are remarkable, because it would be expected that most changes occurred just after the credit crunch in 2008, a period in which tax levies and national allocation rules were subject of intensive debate in the sector. It might be that respondents perceived the changes to have also taken place in the period after 2010, but on the basis of the available research data this explanation can neither be confirmed nor be refuted. Another possible explanation was offered by one of the interviewees, a senior policy officer, who said:

In the first years of the crisis, we could well go on with the building projects that were still in the pipeline. But now we have much less new projects, and are confronted with a much stricter bank lending policy for the projects that we want to realise.

This would mean that the consequences of the economic crisis have been increasingly felt in the years that followed. The most visible shifts are related to the emphasis on commercial activities (strong decrease) and on financial return (strong increase compared to social return).

### Shifts in strategies

**Fig. 8:**
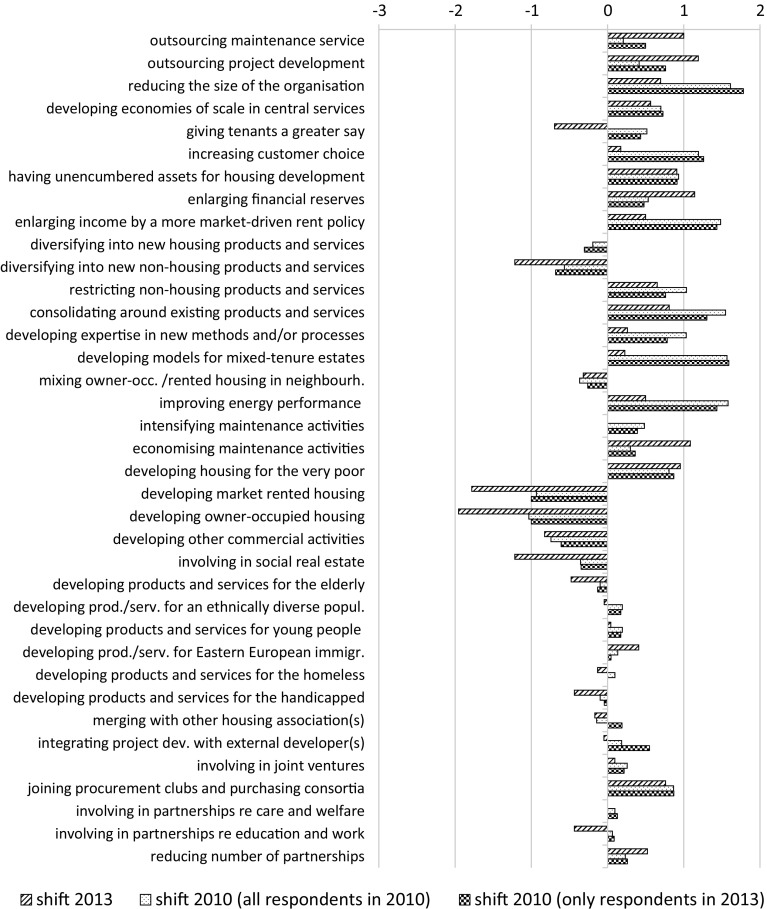
Shifts in importance of strategies reported in 2010 and in 2013

As has been stated in Sect. 4, in both the 2010 and the 2013 survey, respondents were asked to indicate the importance of a number of strategies for the last 3 years preceding the survey and for the subsequent 3 years. The differences in importance between these two periods are depicted for each survey in Fig. 8. A negative value denotes a decreasing importance, while an increasing importance is indicated by a positive value.

As with the strategic priorities, the directions of the shifts are the same for almost all items, implying that the trends identified in the first wave of the research persisted. Nevertheless, the size of the shifts can be very different between the two waves. For instance, increased shifts can be observed regarding outsourcing maintenance service, outsourcing project development, enlarging financial reserves and economising maintenance activities (growing importance), as well as the development of owner-occupied housing, market rented housing and social real estate (diminishing importance). On the other hand, reducing the size of the organisation, improving the energy performance of the housing stock and consolidating around existing products and services, for instance, was seen as more important for the next years than for the last years in both waves. However, the increase in importance in 2010 was bigger than in 2013.

### Future strategic decisions

In the questionnaire, respondents were asked to describe the most important strategic decisions that would be made by their organisation in the coming 3 years. While space was given for stating three decisions, some respondents reported only one or two decisions. The 31 respondents in the first wave mentioned 92 decisions in total, while 65 were noted by the 23 respondents that took part in the second wave. Table 3 presents a categorisation of the reported decisions and, for both waves, the share of each of the categories.

**Table 3:** Share of types of most important future strategic decisions, in 2010 and 2013

Type of decision	Share of reported strategic decisions in the first wave (2010) (%)	Share of reported strategic decisions in the second wave (2013) (%)
Back to core business	11	8
Reorganisation	14	17
Income and financial position	13	11
Shift in markets and target groups	8	8
Energy and sustainability	9	3
Geographical territory	3	2
Return and efficacy	11	9
Desired type of organisation	8	3
Affordability	0	6
Client orientation, relationship with tenants	8	6
Relationship with external parties	2	8
Portfolio policy and building programming	10	17
Remaining	4	3

The table shows that decisions regarding reorganisation, income and financial position, and portfolio policy and building programming were typically seen as the most important strategic decisions, both in the first and the second wave (more than 10 % for each type in both waves). As for the first wave, this is also true for decisions explicitly aiming at a move back to core business and for decisions related to return and efficacy. However, when we compare the respective shares with those of the second wave 2013, a slight decline can be observed.

The table shows considerable decline in the share of decisions regarding energy and sustainability (from 9 to 3 %, which is remarkable given the importance attributed to the topic for the 3 years after the survey, see Fig. 6) and the desired type of the organisation (from 8 to 3 %). Conversely, the share of decisions regarding portfolio policy and building programming (from 10 to 17 %), affordability (from 0 to 6 %) and the relationship with external parties (from 2 to 8 %) increased considerably. In the subsequent interviews the participants partially explained these trends. With respect to the relationship with external parties, several interviewees indicated that their organisations reformulated their role: in the past, housing associations were often in an initiating and financing role, but nowadays they tend to take a more modest role. This can also mean that they have to look for new parties to realise their ambitions. One of the interviewees said:

Innovation remains necessary. This can be done in all kinds of areas: involvement of stakeholders, cooperation in procurement, determining a regional investment agenda, designing and implementing new care arrangements. All under the condition that these activities are explicitly related to the core task of the housing association.

The position of affordability as a “newcomer” in the list of decision types in Table 3 might be rather unusual, as it might imply that housing associations only recently started to assign importance to affordability. Subsequent interviews, nevertheless, indicate a growing awareness of the subject, caused by lower household income (mostly due to unemployment), the need to raise rents (to generate sufficient income) and new government regulations facilitating further rent increases.

## Conclusions

As a result of political and economic developments, Dutch housing associations have been reducing their field of operations, focussing much more on traditional social housing tasks, following over a decade of expansion of activities that lasted until around 2008. The second wave of the research conducted in 2013/2014 shows that the surveyed housing associations have continued and will continue the retreating movement that also became apparent in the first wave in 2010/2011. Our inquiry into the current values shows a greater entrepreneurial orientation combined with a much stronger business ethos than in 2010. This shift points towards an increased focus on budget savings and efficiency. It does not, however, mean an increased focus on commercial activities: the statements made about activities indicate a reduction in non-housing services and “commercial” development and a stronger focus on the provision of rental housing for low-income households. Looking at the strategies, the general picture in 2013 is the same as in 2010. Again, a stronger emphasis on reduction in the number of staff and in maintenance costs can be observed, reinforcing the expectation that increasing business efficiency has gained even more importance now than in previous years. When we compare the priorities of the surveyed housing associations in 2010 with 2013 and the expectations for 2016, we see that the priorities in 2016 and 2013 are well aligned and that the main shifts have taken place before 2013.

The results of our analysis confirm our expectations that the retreat of Dutch housing associations continued after 2010. Contrary to our expectations, however, the most significant shifts have not taken place just after the onset of GFC, but later, in the period between the two research waves. Our findings also indicate that Walker’s (2000) scenario for the UK context, suggesting that on-going managerial and external pressures on social housing organisations are likely to lead to the domination of a property-based approach, emphasising business efficiency, is currently taking shape in the Netherlands. In terms of the classification developed by Gruis (2008), we see a continuous shift from a Prospector strategy towards a Defender strategy. In other words, housing associations are becoming increasingly similar to the archetype of Social Housing Investors.

In terms of the “tension field” (Mullins et al. 2014) between government, market and community (discussed in Sect. 2), we observe a movement from market towards government, first because of the less favourable market conditions (push factor), second because of government policies (pull factors). While government policies in the 1990s aimed at “pushing away” the housing associations towards market and also community, current policies try to draw these organisations closer to the government.

Gruis’ model, especially the distinction between defenders and prospectors, has proven useful in identifying and describing the developments in the Dutch non-profit sector. Nevertheless, because current developments lead to a concentration of housing associations in just one category, namely that of the social housing manager, the typology may become less relevant in the near future. However, the study did not produce material for an adaptation of the model. From a theoretical perspective though, it may be interesting to link the developments and drivers to other theoretical frameworks as well, such as the process of isomorphism. Anheier (2005) suggests that non-profit organisations are subject to three mechanisms of institutional isomorphic change as distinguished by Powell and DiMaggio (1991):

- coercive isomorphism, which appears as a reaction to direct or indirect pressures;
- mimetic isomorphism, which occurs when organisations model themselves after other organisations that are perceived as successful;
- normative isomorphism, which derives from professional norms and standards that may be dominant in certain sectors.

Although these shapes of isomorphism are not mutually exclusive, it would be interesting to see whether some shapes are more dominant now than in the past. For example, it can be expected that coercive isomorphism has gained dominance in recent years (with housing associations collectively adopting a defender strategy), while mimetic isomorphism was more dominant in the past period of diversification (when more and more housing associations where adopting behaviour of prospectors). Further in-depth research in the underlying values and drivers could be conducted to validate this assumption.

From a wider societal perspective, it is relevant to see whether and which social problems will occur or will be reinforced by these developments. One of the main points of concern is the tension between the necessity to increase rental income on the one hand and growing concerns about affordability on the other, combined with a more stringent focus on low-income households. It is questionable whether the current political pressure on housing associations to raise rents and, at the same time, to concentrate on low-income households can be sustained in the longer term. Furthermore, several studies have pointed out the risk of a substantial shortage of housing affordable for middle-income households in the Netherlands (e.g. Kromhout et al. 2010). While housing associations are pushed to focus more and more on the lower-income households, it remains highly questionable whether private investors will take over their role in the market for middle-income households.

Another point of concern is the effect that the reduction of community investments as well as physical restructuring activities will have on vulnerable neighbourhoods. A further challenge will be to combine business efficiency with a satisfying level costumer support and service. Monitoring the outcomes of the shifting priorities will reveal the extent to which the housing associations are able to cope with these challenges.

Finally, it will be interesting to identify a divergence or convergence in non-profit housing sectors in different countries. Are more or less general neo-liberal policies and economic developments leading to similar trajectories? While there is ample evidence pointing towards a shift away from public housing provision (e.g. Gibb 2002; Malpass and Victory 2010; Blessing 2014), it is largely unknown whether this trend leads to convergence in the strategies of individual housing providers. If the developments in the Netherlands are compared with those in the UK and Australia (see e.g. Mullins et al. 2014), the provisional answer must be negative. More specifically, contrary to the retreat in the Netherlands, these countries show a diversification into private sector activities. Although, at different times, the financial role of the government has diminished in all three countries, the Dutch case shows that this does not necessarily coincide with a weaker regulatory role.
